# Cryptotanshinone Inhibites Bladder Cancer Cell Proliferation and Promotes Apoptosis via the PTEN/PI3K/AKT Pathway

**DOI:** 10.7150/jca.31422

**Published:** 2020-01-01

**Authors:** Yadong Liu, Fanlu Lin, Yaodong Chen, Rui Wang, Jiannan Liu, Yinshan Jin, Ruihua An

**Affiliations:** 1 Department of Urology. The First Affiliated Hospital of Harbin Medical University, Harbin, Heilongjiang, 150001, People's Republic of China;; 2 Department of Urology. Linyi Central Hospital, Linyi, Shandong, 276400, People's Republic of China;; 3 Department of ultrasonic imaging, First Hospital of Shanxi Medical University, Taiyuan, 030001, China.

**Keywords:** cryptotanshinone, bladder cancer, proliferation, apoptosis, PTEN/PI3K/AKT.

## Abstract

Cryptotanshinone (CTT), extracted from the root of *Salvia miltiorrhiza Bunge* (Danshen), exhibits activities against a variety of human cancers *in vitro* and* in vivo*. The purpose of this study was to investigate the potential inhibitory effect of CTT on bladder cancer. In this study, we found that CTT inhibited bladder cancer cell proliferation, migration, and invasion and promoted apoptosis. In addition, CTT modulated the expression of proteins via the PI3K/AKT pathway, and the inhibition of PI3K/AKT signalling was due to induction of PTEN expression. Taken together, the results of the present study demonstrated the anticancer effect of CTT on bladder cancer cells, which might be associated with the downregulation of PI3K/AKT/mTOR and NF-κB signalling pathway proteins, and this inhibition was mediated by the induction of PTEN.

## Introduction

Bladder cancer is the 7th most common tumour worldwide in males, while it is the 17th most common tumour in females and is one of the most deadly urothelial malignancies 1. Almost three-quarters of newly diagnosed bladder cancer cases are non-muscle-invasive bladder cancer (NMIBC), which can be treated with transurethral resection of the tumours followed by intravesical instillation of chemotherapeutic drugs 2. However, bladder cancers have a high recurrence rate, and it is estimated that 25% of patients with NMIBC develop muscle-invasive bladder carcinoma following treatment 3. Once bladder cancer develops into the invasive stage, the prognosis and treatment become limited 4. Although various anticancer agents, such as radiotherapy, chemotherapy and hyperthermia, have been shown to inhibit tumour recurrence and progression, their toxic side effects and limited response rate remain major clinical problems 5-7. Therefore, novel or alternative agents with fewer side effects for bladder cancer patients are urgently required.

Numerous studies have confirmed that the phosphatidylinositol-3 kinase (PI3K)/Akt (protein kinase B)/mammalian target of rapamycin (mTOR) pathway plays an important role in cell proliferation, metastasis and angiogenesis in bladder cancer 8, 9. Moreover, studies have demonstrated that blocking the PI3K/Akt/mTOR pathway can be a potential therapeutic strategy 10. For example, an mTOR inhibitor called everolimus could significantly prevent the growth of bladder carcinoma *in vitro* and *in vivo* 11. Moreover, NVP-BEZ235, a dual PI3K and mTORC1/2 inhibitor, exhibits strong antitumour activity in bladder cancer studies 12. Therefore, the PI3K/Akt/mTOR pathway might be a potential target for the treatment of bladder cancer 13.

Cryptotanshinone (CTT) is extracted from the root of *Salvia miltiorrhiza Bunge* (Danshen) and has numerous pharmacological effects, including antioxidant, anti-hyperlipidaemia, anti-inflammation and anti-diabetes effects 14, 15. Moreover, numerous studies have shown that CTT exhibits activities against a variety of human cancers *in vitro and in vivo* and can be used for the management of human leukaemia, colorectal cancer, pancreatic cancer, breast cancer, prostate cancer, liver cancer, and gastric carcinoma 16-18. However, the potential effect of CTT on bladder cancer and its mechanism remain unknown.

## Materials and Methods

### Reagents and chemicals

**Figure 1:**
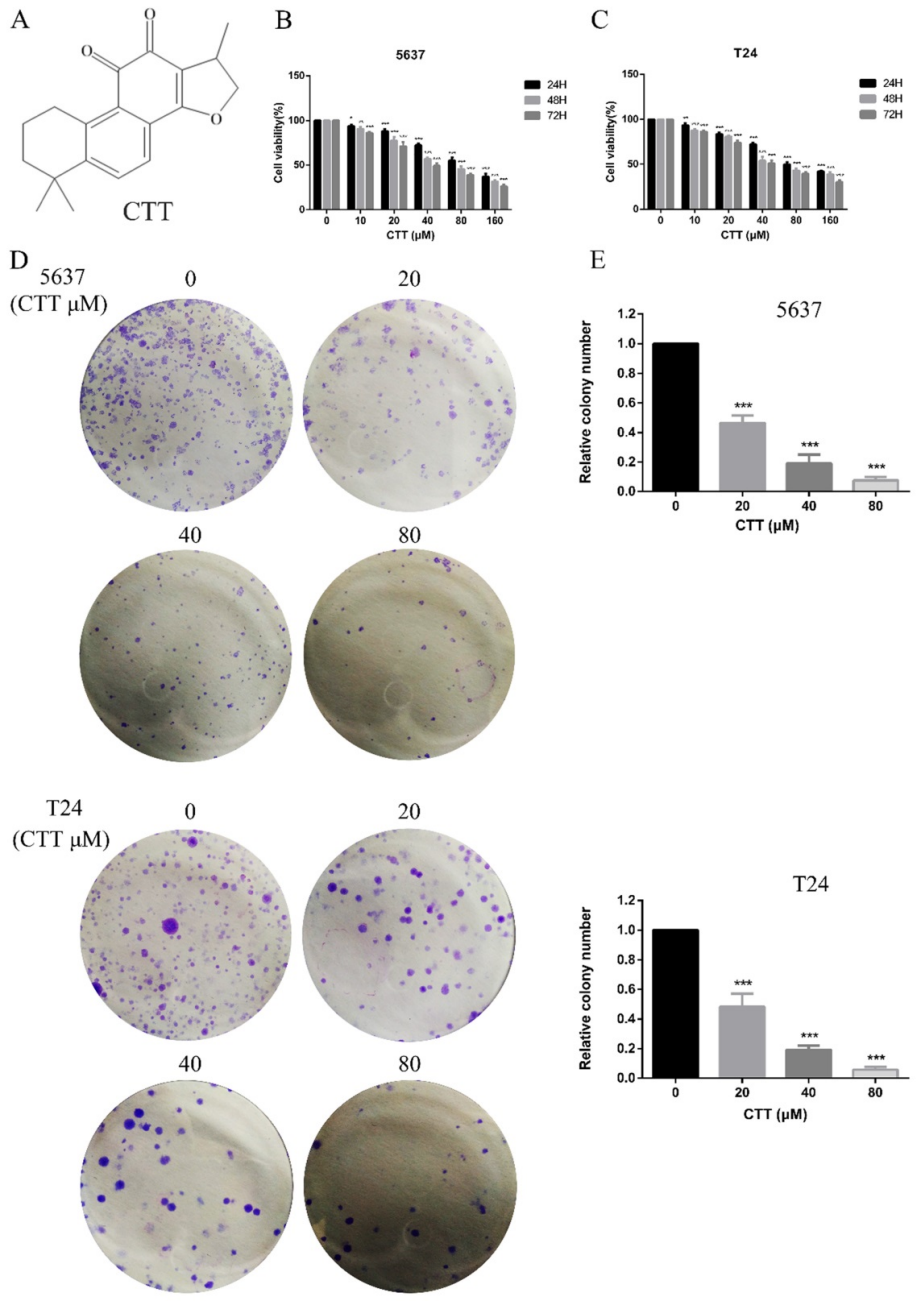
** Effect of cryptotanshinone on the viability of bladder cancer cells.** (A) Chemical structure of CTT. (B, C) Bladder cancer cell lines (5637, T24) were treated with various concentrations of CTT (0, 10, 20, 40, 80 or 160 μM) for 24, 48 and 72 h. Cell viability was measured by a CCK-8 assay. (D) Anti-proliferation effect of CTT on 5637 and T24 cells by colony formation assay. (E) Histograms show the colony numbers of 5637 and T24 cells. The data shown are representative of at least three independent experiments. ^*^ P < 0.05, ^**^ P < 0.01, ^***^ P < 0.001, compared with the control group.

5637 and T24 cells were donated by Xu Qingquan from the Urology Laboratory of People's Hospital affiliated with Peking University Medical College. CTT was purchased from Shanghai Aladdin Biochemical Technology Co., Ltd. (Shanghai, China), and purity was assessed by HPLC (˃98%). CTT was dissolved in methanol at 80mmol/L stock concentration and stored at -20°C. CTT was then dissolved and diluted in culture medium at the appropriate concentrations in all cell experiments. The CTT structure is shown in Figure 1. RPMI-1640 medium and foetal bovine serum (FBS) were obtained from HyClone (Thermo Fisher Scientific, Waltham, MA, USA). Cell Counting Kit 8 (CCK-8 Kit) was purchased from Dojindo Molecular Technologies. Propidium iodide (PI) was purchased from Sigma-Aldrich Chemical Company (St. Louis, MO, USA). Rabbit antibodies against mouse cleaved caspase-9, cleaved caspase-3, Bcl-2, Bax, matrix metalloproteinase-2 (MMP-2), matrix metalloproteinase-9 (MMP-9), vimentin, E-cadherin, PI3K, phosphatase and tensin homologue (PTEN), p-mTOR (Ser2448), p-AKT (Ser473), AKT, NF-κB and β-actin were obtained from Cell Signaling Technology (Danvers, MA, USA). Primary antibodies for detecting Bad and Bcl2L2 were all purchased from Abcam (Cambridge, UK). The secondary antibodies, including HRP-conjugated AffiniPure goat anti-rabbit IgG and HRP-conjugated AffiniPure goat anti-mouse IgG, were purchased from ZhongShan Golden Bridge Bio Co., Ltd. (Beijing, China).

### Cell culture

Two human bladder carcinoma cell lines, 5637 and T24, were grown in RPMI-1640 containing 10% (v/v) FBS, 100 U/mL penicillin and 100 μg/mL streptomycin. Cells were cultured at 37°C in a 5% CO_2_ humidified environment.

### Cell viability assay

The proliferation and cytotoxicity of cells were determined using a CCK-8 assay (Dojindo Molecular Technologies, Beijing, China). A total density of approximately 5 × 10^3^ cells/well 5637 and T24 cells was seeded in 96-well plates for 24 h. The cells were treated with different concentrations of CTT in 200 μL per well and incubated at 37°C, 5% CO_2_ for 24, 48, and 72 h. Subsequently, CCK-8 was added to each well and incubated in a high-humidity environment at 37°C and 5% CO_2_ for 1 h. The absorbance was measured at a wavelength of 450 nm.

### Apoptosis assay

5637 or T24 cells were plated in 6-well plates at a density of 5×10^5^ cells/well and incubated with 0, 20 or 40 µM CTT for 48 h at 37°C. Flow cytometry (BD Biosciences, Franklin Lakes, NJ, USA) and Hoechst 33258 (Wanleibio, Shenyang, China) staining were used to measure the apoptosis of the two cell lines. The relative amount of Annexin V-fluorescein isothiocyanate-positive/propidium iodide-negative cells were detected using an Apoptosis Detection Kit I (BD Biosciences) and analysed using FlowJo 7.6.1 (BD Biosciences).

### Colony formation

T24 or 5637 cells were seeded into 6-well plates at a density of 1×10^5^ cells/well in 2 mL of medium. After treatment with various concentrations of CTT for 48 h, the cells were collected and diluted in fresh medium in the absence of CTT and then reseeded into 6-well plates at a density of 1×10^3^ cells/well. Following incubation for 8 days in a 37°C humidified incubator with 5% CO_2_, the formed colonies were fixed with 10% formaldehyde, stained with 0.1% crystal violet and counted. Cell survival was calculated by normalizing the survival of the control cells to 100%.

### Cell invasion assay

For the invasion assay, 50 µL of Matrigel matrix was added to the upper surface before adding cells. After four hours, cells were collected and resuspended in RPMI, and then, 200 µL of the cell suspension (10^5^ cells) was placed in transwell chambers (CORNING, Corning, NY, USA) in 24-well plates 48 h after CTT treatment. Then, 500 µL of RPMI medium supplemented with 10% FBS was added to the lower chamber. After culture for 24 h, the cells in the upper layer were wiped away using cotton swabs, while the cells in the lower layer were fixed with 4% paraformaldehyde and stained with 0.1% crystal violet (Beyotime Institute of Biotechnology, Shanghai, China) for 40 min. The cells in the lower compartment were those that had invaded. We counted five randomly selected fields to calculate the average cell number in triplicate.

### Wound-healing assay

T24 or 5637 cells were plated in 6-well cell culture plates for 24 h in 2 mL of RPMI medium containing 10% FBS. After the cells formed a confluent monolayer, the cells were scraped with a pipette tip, and the cells were cultured in medium containing different concentrations of CTT (0, 2, 4, or 8 µM). After 24 h, the cell migration distance was examined. Measurements of the wound width were made at the beginning of the experiment and 24 h later. Measurements were made at 20× magnification using an ocular grid.

### Gene silencing

Human 5637 cells were seeded into 6-well plates (2.5×10^5^ cells/well) and allowed to adhere for 24 h before treatment with CTT. Using Lipofectamine 2000 (Invitrogen, Groningen, Netherlands), the cells were transfected with siRNAs directed against human 5637 cells (PTEN siRNA; GenePharma, Shanghai, China) or with non-targeted control siRNAs (NC siRNA; GenePharma).

### Immunofluorescence

After treatment with different concentrations of CTT, bladder cells were washed with cold phosphate-buffered saline (PBS) twice and fixed in 4% paraformaldehyde for 30 min. Following fixation, cells were then washed with PBS three times for 15 min and then permeabilized with 0.5% Triton X-100 for 20 min at room temperature. Cells were then washed with PBS three more times before incubating with primary antibodies against p-Akt and PTEN (1:100 dilution) at 4°C. The next day, the slides were stained with corresponding FITC-conjugated secondary antibodies for 2 h at room temperature. The nuclei were then stained with the DNA-binding dye 4′,6-diamidino-2-phenylindole dihydrochloride (DAPI) for 5 min and examined by fluorescence microscopy (Leica DMI3000B, Leica Microsystems, Wetzlar, Germany).

### Western blot analysis

The treated cells were washed with PBS and lysed with RIPA buffer, phosphatase inhibitors and PMSF at a ratio of 100:10:1 to obtain total protein for Western blotting. Equal amounts of proteins were separated by SDS-PAGE and transferred to a polyvinylidene fluoride membrane. After transfer, the polyvinylidene fluoride membrane was blocked in 5% fat-free milk/1 × TBS/0.1% Tween-20 for 1 h at room temperature and then incubated with primary antibodies overnight at 4°C. Then, the membrane was washed with 1× TBS/0.1% Tween-20 before incubation with secondary antibodies for 1 h at room temperature. The immunoreactivity was detected using an Odyssey Infrared Imaging System. The bands were quantified by measuring the band intensity for each group 19.

### Animal studies

BALB/c mice (4-6 weeks old) were divided into a vehicle-treated control group (n=6) and a CTT-treated group (n=6). All studies involving mice were carried out under protocols approved by the Animal Research Reporting of *In vivo* Experiments (ARRIVE) guidelines. Mouse handling was performed in accordance with an animal study protocol approved by the National Cancer Institute Animal Care and Use Committee. The mice were maintained under a standard 12-h light/12-h dark cycle, with water and chow provided ad libitum. CTT was dissolved in culture medium and then diluted in PBS buffer (v/v=1:1000). CTT (0 or 25 mg/kg body weight) was given to mice by a single intraperitoneal injection every 2 days for 3 weeks. After euthanasia by CO_2_ asphyxiation, the gallbladder, liver, duodenum, jejunum, ileum, and colon were harvested, and a small section of each tissue was excised for histological analysis. All samples were stored at -80°C until analysis. The tumour size was measured in two orthogonal directions using callipers every three days, and the tumour volume (mm^3^) was estimated using the equation: length×(width)^2^ × 0.5. Four weeks later, the mice were sacrificed, and the tumours were resected.

### Statistical analysis

All data are expressed as the mean ± SD of three independent experiments. Statistical significance was determined using a Student's t-test or ANOVA. A P value of less than 0.05 was considered significant. A detailed description of the methods can be found in the online Supporting Information.

## Results

### CTT inhibited bladder cancer cell proliferation and promoted apoptosis of 5637 and T24 cells

**Figure 2:**
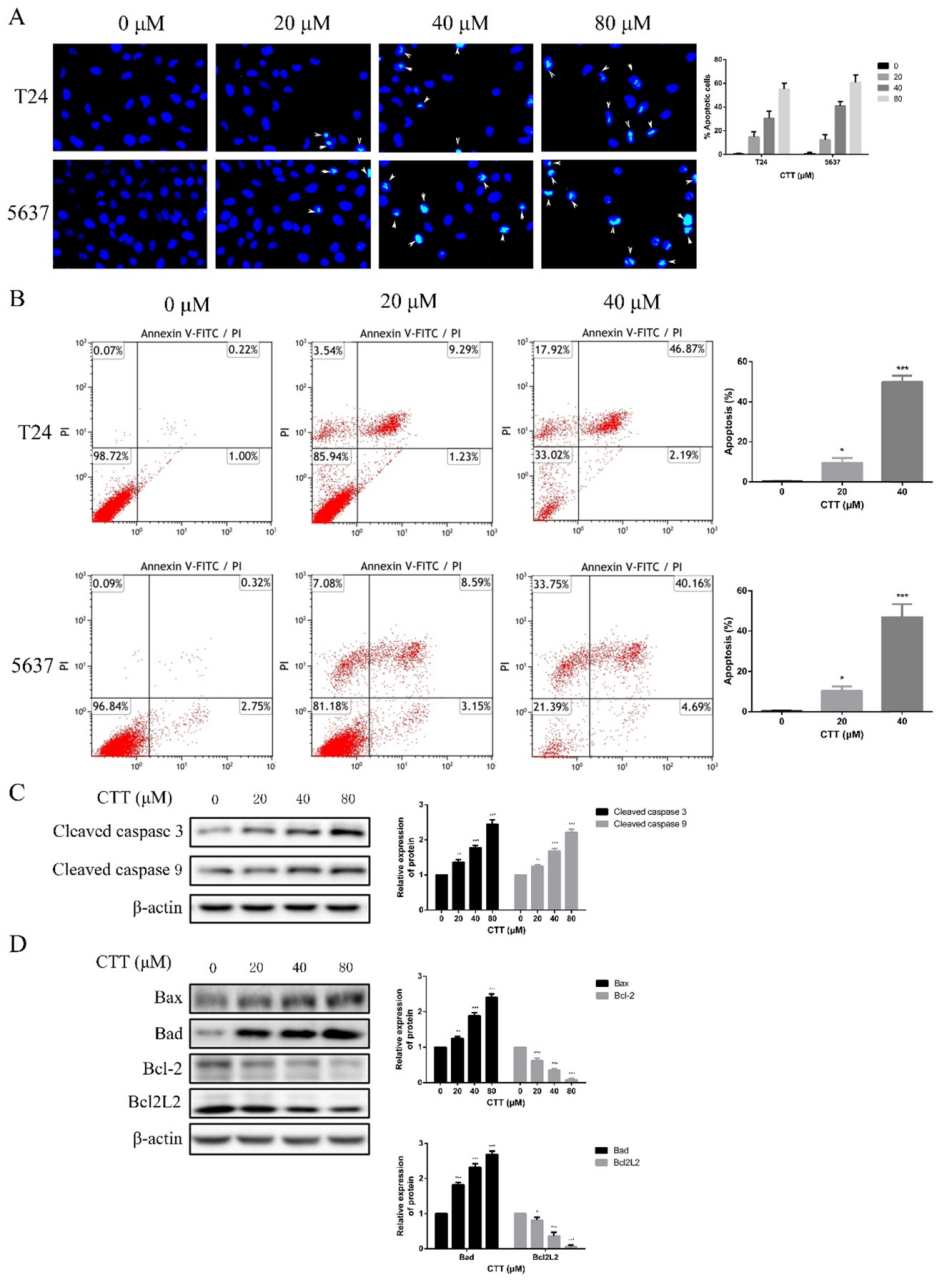
** Effect of CTT on apoptosis of bladder cancer cells *in vitro*.** (A) Hoechst 33258 staining was used to observe the morphology of the cell nucleus in the bladder cancer cell lines 5637 and T24 incubated with CTT (×100 magnification). (B) Bladder cancer cells were incubated with a gradient of CTT concentrations for 48 h. Representative flow cytometry histograms of assays performed to detect apoptosis. (C) Representative histograms for the apoptotic rate in 5637 and T24 cells. (D) Respective expression of cleaved caspase 3, cleaved caspase 9, Bcl-2, Bad, Bcl2L2 and Bax was analysed by Western blotting with β-actin as the loading control. (E) The density of each band was examined for a statistical comparison of protein expression levels normalized to the β-actin level.

To investigate the anti-apoptotic effect of CTT (Figure 1A), 5637 and T24 cells were treated with different concentrations of CTT (0-160 μM) for 24, 48 and 72 h, and cell viability was detected by the CCK-8 assay. CTT suppressed the viability of 5637 and T24 cells in a dose- and time-dependent manner (Figure 1B, C). Besides, the results also showed that methanol itself had little effect on cell activity (T24 and 5637) at 48 h (Additional file 1: Figure S1). The addition of methanol diluted 1000 times had no effect on the expression of p-Akt and PTEN protein (Additional file 2: Figure S2). In addition, the colony formation of 5637 and T24 cells was reduced significantly by CTT in a dose-dependent manner (Figure 1D). Furthermore, to investigate the anti-apoptotic effect of CTT, 5637 and T24 cells were stained with Annexin V and propidium iodide (PI), followed by flow cytometry (FCM) analysis. Figure 2A and B shows that CTT promoted the apoptosis of 5637 and T24 cells in a dose-dependent manner. To further investigate this finding, Western blotting was performed to detect the expression of important signalling proteins involved in apoptosis. The data showed that the expression level of Bax and Bad increased, the expression level of Bcl-2 and Bcl2L2 decreased, and the activity of caspase-3 and -9 was enhanced, which plays a crucial role in the progression of apoptosis (Figure 2D, E). Taken together, these findings indicate that CTT may inhibit proliferation and promote apoptosis of bladder cancer cells.

### CTT inhibited bladder cancer cell migration and invasion

**Figure 3:**
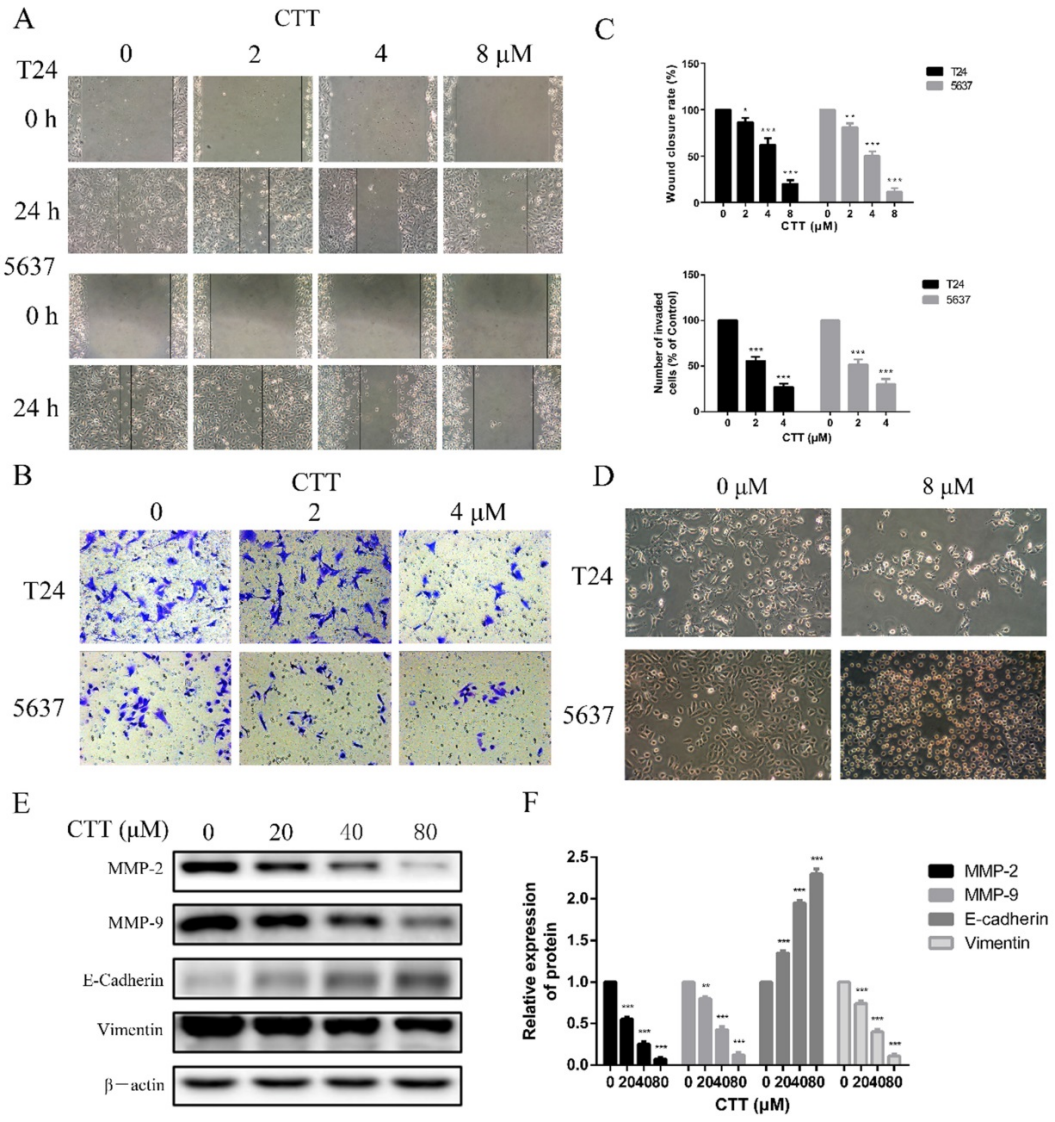
** Wound healing assay and transwell assay were used to evaluate the effect of CTT on migration and invasion of bladder cancer cells.** (A) Cells were treated with different concentration of CTT and representative images from wound healing assays were acquired at 0 and 24 h. (B) The invasion ability of 5637 and T24 cells was also quantified by Transwell assays. (C) Histograms exhibited wound closure rate and the number of invaded cells. (D) The morphological changes of T24 and 5637 cells in the presence of CTT. (E) Role of CTT on the protein levels of MMP2, MMP9, E-cadherin and Vimentin. The relative protein levels were normalized to the β-actin level. The quantitative results were expressed as the means± SD of three experiments. ^*^ P < 0.05,^ **^ P < 0.01, ^***^ P < 0.001, compared with control group.

To investigate the effect of CTT on bladder cancer cell migration and invasion, wound-healing and transwell assays were performed using 5637 and T24 cells. CTT inhibited 5637 and T24 cell migration in a dose-dependent manner (Figure 3A). Consistently, as shown in Figure 3B, the invasion ability of 5637 and T24 cells was also dose-dependently reduced by CTT in a transwell assay.

To further verify the anti-migration and anti-invasion effects of CTT, we performed experiments to investigate the inhibitory effects of CTT on the epithelial-to-mesenchymal transition (EMT), a critical event in cell invasion and migration. We initially detected that bladder cancer cells exposed to 0 or 8 μM CTT for 24 h exhibited an altered morphology from a fibroblast-like mesenchymal phenotype to a round epithelioid phenotype (Figure 3D). In addition, we further assessed the expression levels of migration-related proteins (E-cadherin, vimentin, MMP2 and MMP9), which are responsible for tissue remodelling and degradation of the extracellular matrix (ECM), to verify these effects. As shown in Figure 3E, CTT inhibited the expression of MMP2 and MMP9 in a dose-dependent manner. These findings indicated that CTT inhibited bladder cell migration and invasion.

### CTT modulated the expression of proteins in the PI3K/AKT pathway

**Figure 4:**
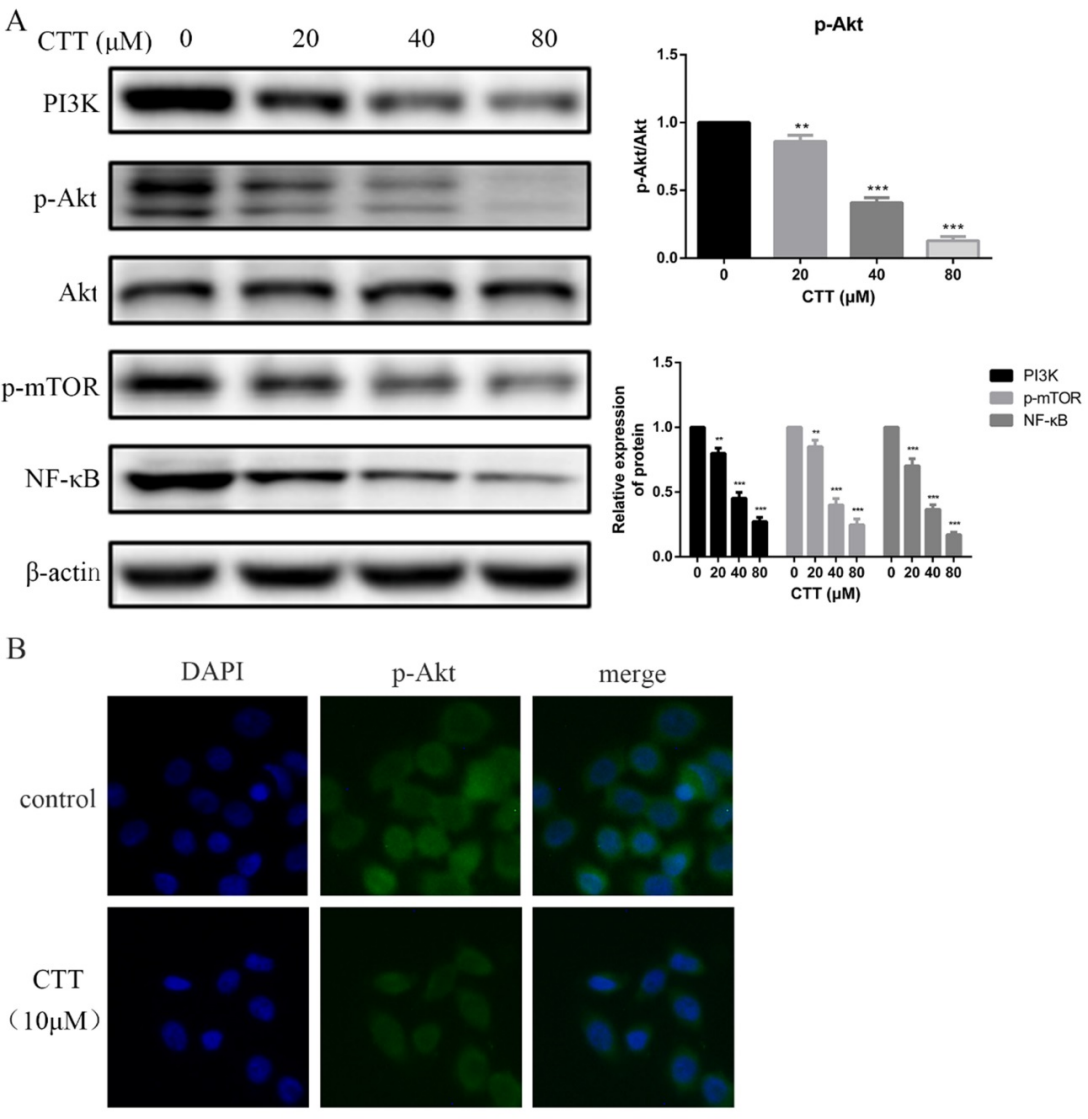
** CTT inhibited the PI3K/Akt and mTOR, NF-κB signaling pathways.** (A) After treatment with different concentrations of CTT, images of protein expression of PI3K, p-Akt, p-mTOR and NF-κB were determined by Western blot. β-actin was used as a loading control. (B) Following treatment with or without CTT for 48 h, the expression of p-Akt in T24 cells was assessed by immunofluorescence (× 200). Each value represented the mean ± SD for triplicate samples. ^*^ P < 0.05, ^**^ P < 0.01, ^***^ P < 0.001, compared with control group.

To investigate whether the PI3K/AKT pathway was involved in the antitumour effects of CTT, the expression levels of proteins involved in the PI3K/AKT pathway were explored using Western blots. The data showed that the protein expression of PI3K, p-AKT, p-mTOR and NF-κB in the treatment group was decreased compared with that of the control group after 48 h (Figure 4A). Consistently, an immunofluorescence assay also demonstrated a significant decrease in p-Akt due to CTT (Figure 4B).

### Inhibition of PI3K/AKT signalling was due to the induction of PTEN expression

**Figure 5:**
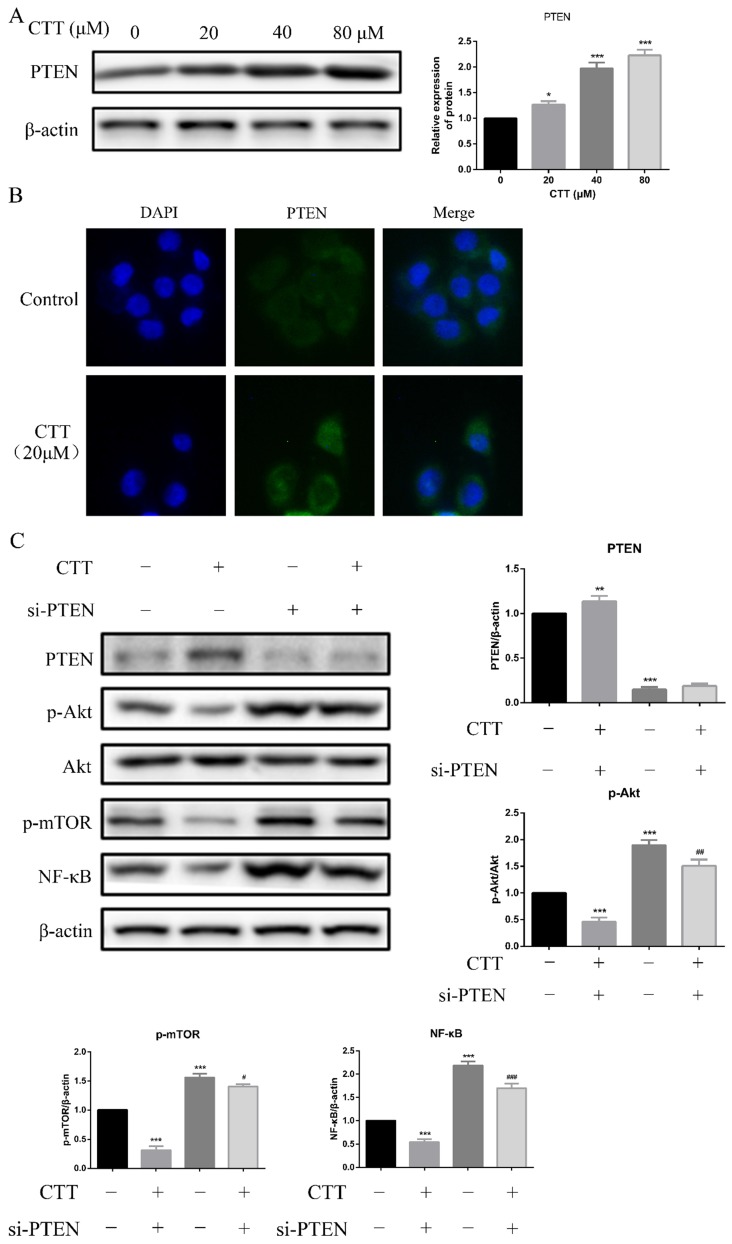
** CTT upregulated PTEN expression *in vitro*.** (A) Images of protein expression of PTEN was detected by Western blotting. (B) Representative immunofluorescence image images of expression of PTEN were evaluated at 0 and 24 h (× 200). (C) 5637 cells were treated with CTT in presence or absence of the PTEN-siRNA, followed by Western blot analysis of the expression of PTEN, p-Akt, p-mTOR and NF-κB proteins. The results were shown as the mean ± SD of three experiments. ^*^ P < 0.05, ^**^ P < 0.01, ^***^ P < 0.001, compared with control group.

Studies have demonstrated that PTEN deficiency is the most common genetic change in bladder cancer; thus, we hypothesized that induction of PTEN was a reasonable explanation for the CTT-induced PI3K/AKT signalling pathway inhibition. As expected, Western blot data showed that PTEN expression was increased in 5637 cells following 24 h of CTT treatment (Figure 5A). In addition, an immunofluorescence assay confirmed the increased expression of PTEN due to CTT (Figure 5B). These findings suggested that CTT-mediated PI3K/AKT pathway inhibition was due to the upregulation of PTEN in bladder cancer cells.

Furthermore, we transfected specific PTEN-siRNA plasmids into 5637 cells, followed by Western blot assays. Figure 5C shows that this manipulation upregulated the expression of p-Akt, p-mTOR, and NF-κB. Collectively, our results demonstrated that in 5637 cells, the antitumour activity of CTT depended on the upregulation of PTEN.

### *In vivo* effects of CTT on bladder cancer

**Figure 6:**
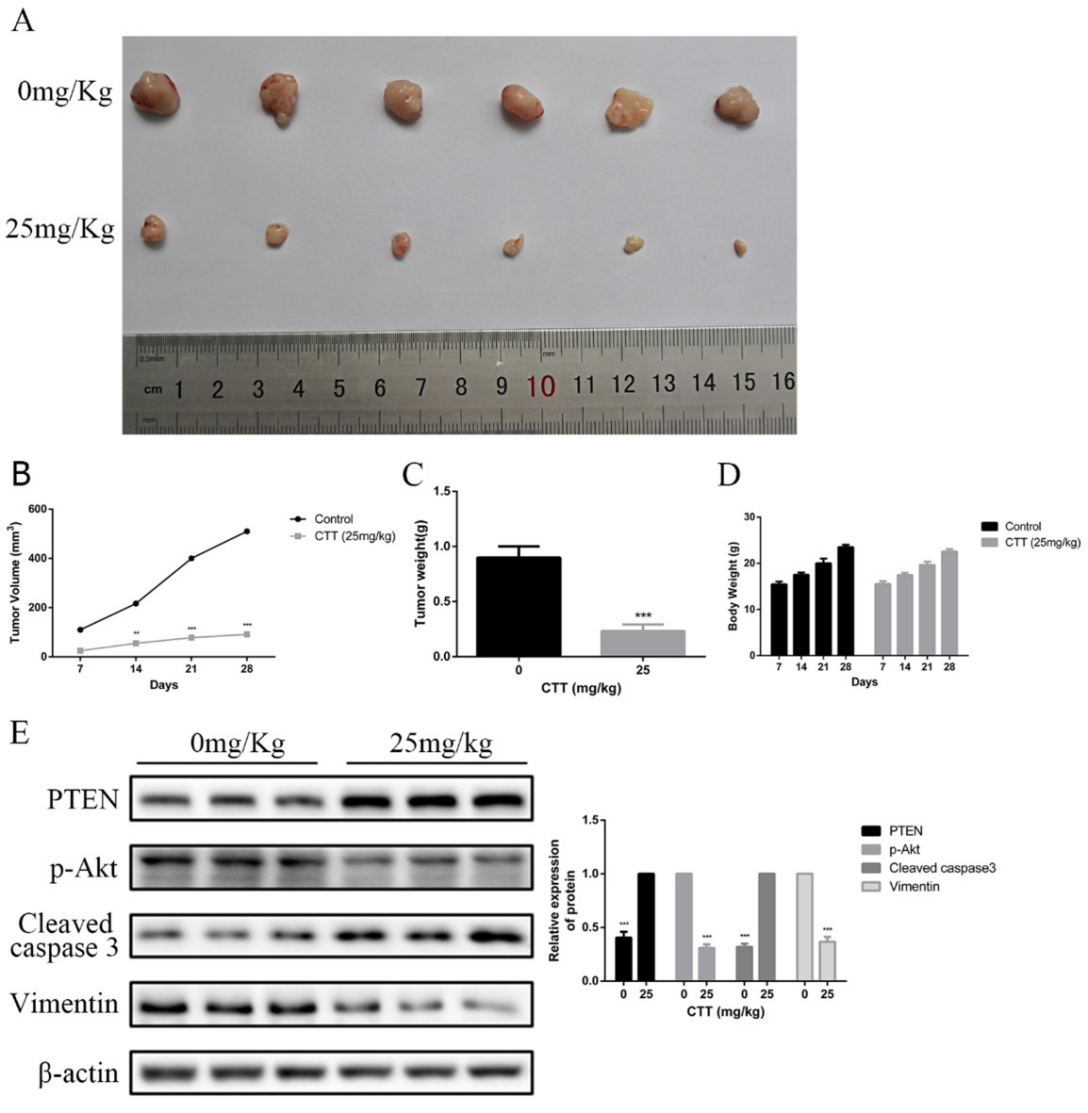
** CTT suppressed bladder cancer growth *in vivo*.** (A) Image showing the inhibition of a xenografted bladder tumour in nude mice treated with CTT. (B) Tumour volumes were calculated. (C) Tumour weights were measured. (D) Body weights are shown. (E) PTEN, p-Akt, cleaved caspase-3 and vimentin protein levels were quantified. The results are from at least three independent experiments. ^*^ P < 0.05,^ **^ P < 0.01, ^***^ P < 0.001, compared with the control group.

To investigate the *in vivo* effects of CTT, a series of experiments were performed in nude mice. Mice subcutaneously implanted with 5637 cells were treated with a vehicle control or CTT at 25 mg/kg every 2 days for 3 weeks after xenografts were established and had grown to a mean size of 90 mm^3^. As shown in Figure 6A, compared with the control group, the administration of CTT in mice significantly inhibited tumour growth. Figure 6B indicates that the mean weight of tumours in the control group was 0.72 g, whereas that of tumours in the CTT-treated group was approximately 0.31 g. In addition, Figure 6C shows the volume change over the experimental period. We also evaluated the broad toxicity of the therapeutic drugs. As Figure 6D shows, CTT administration during the indicated periods led to no apparent toxicity-related events or significant body weight changes.

**Figure 7:**
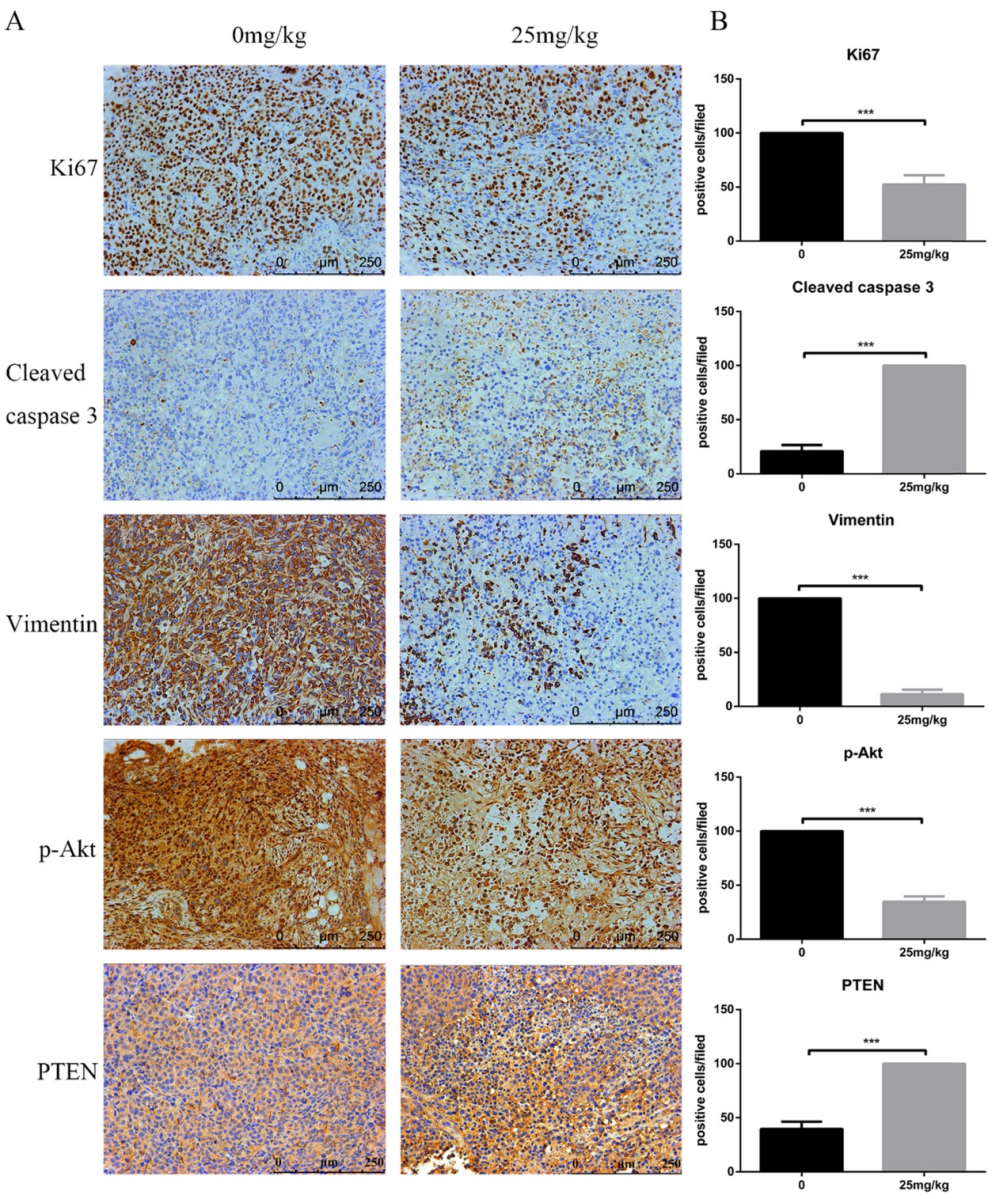
** Effects of CTT on cell proliferation, apoptosis, and invasion in bladder tumours.** Representative immunohistochemistry images of bladder tumour tissues (× 200). Level of Ki-67, vimentin, p-Akt were decreased, while PTEN and cleaved caspase-3 were increased, in the CTT-treated group. The values are the mean ± SD of at least 3 independent experiments. ^*^ P < 0.05, ^**^ P < 0.01, ^***^ P < 0.001, compared with the control group.

At the end of the experiment, PI3K/Akt-related proteins in tumour tissues were detected via Western blot. Figure 6E shows the downregulation of p-Akt and vimentin but the upregulation of cleaved caspase-3 and PTEN. Moreover, immunohistochemistry assays confirmed a decrease in Ki-67 and vimentin, whereas an increase in PTEN and cleaved caspase-3 was observed. In line with these results, analysis of the *in vivo* experiments revealed that CTT exhibited an extreme antitumour effect on bladder cancer, and the inhibition was likely due to the inhibition of the PI3K/Akt pathway induced by the upregulation of PTEN (Figure 7).

## Discussion

Multiple genes, processes and stages are involved in the development of bladder cancer 20. Abnormalities in apoptosis and cell EMT changes have been closely correlated with bladder cancer progression 21. Various treatment agents often inhibit tumour cells by promoting apoptosis, blocking cell cycle arrest or inhibiting cell EMT changes 22, 23. In this study, we found that a new Akt inhibitory agent, CTT, induced significant decreases in cell proliferation and suppressed the migration and invasion of bladder cells. In addition, we found that the expression level of PTEN increased after CTT treatment, which indicated that PTEN was a key modulator of the induction of PI3K/Akt signalling 24. Inhibition of PTEN expression reversed the inhibitory effects of CTT. Moreover, in tumour-bearing mice, we showed a high propensity of CTT to inhibit proliferation and metastasis of bladder cells without apparent toxicity-related events or significant body weight changes.

CTT, a natural active element extracted from *Salvia miltiorrhiza Bunge* (Danshen), has antitumour activity towards a broad spectrum of cancer types, including breast cancer, liver cancer and malignancies of the alimentary tract, and it is associated with a low level of toxicity and is well tolerated by patients with cancer 25-28. Consistent with other reports, this study showed that CTT exhibited high levels of anticancer activity on 5637 and T24 bladder cancer cells by suppressing survival and promoting apoptosis 29. In addition, we also found that CTT could weaken bladder cancer cell migration and invasion by suppressing cell EMT changes.

However, the mechanism of CTT inhibition of bladder tumours is not fully clear. The PI3K/Akt signalling pathway is an important intracellular mediator that is critical for the regulation of cell survival and proliferation 30, 31. Numerous studies have demonstrated that aberrant activation of the PI3K/Akt pathway is involved in the pathological process of bladder cancer, and its inhibition has become a useful therapy for bladder cancer 32, 33. Importantly, Kim et al. demonstrated that CTT induced apoptotic cell death and cell cycle arrest through the inhibition of the PI3K/AKT/GSK3B signalling pathway in human lung cancer cells 34. Additionally, Ke et al. observed that treatment of cholangiocarcinoma cells with CTT could induce apoptosis by suppressing PI3K/AKT/NF-κB signalling pathway 35. As Zhang et al. reported that CTT can inhibit the proliferation and migration of lung cancer cells, which was attributed to the IGF-1R-mediated phosphoinositide 3-kinase/AKT pathway 36. Moreover, Liu et al. revealed that CTT suppresses the proliferation, growth, invasion, inflammation and angiogenesis in colon cancer cells through regulating the PI3K/AKT/mTOR pathway 37. For this reason, we performed experiments to assess the inhibitory effects of CTT due to the PI3K/Akt pathway. Western blotting analyses revealed that CTT treatment significantly reduced expression of the PI3K protein. Although CTT did not regulate the expression of total Akt, it significantly suppressed its phosphorylation. Furthermore, CTT suppressed the expression of NF-κB, which mediates the effects associated with anti-apoptotic protein subcellular localization and cell viability, and mTOR, which is associated with cell behaviour, in 5637 cells. We also found that CTT inhibited the migration of 5637 bladder cancer cells though the PI3K/Akt signalling pathway. These results indicate that the inhibitory effect of CTT on bladder cancer depends on the suppression of the PI3K/Akt/mTOR and NF-κB pathways.

PTEN expression is reduced in various tumour types, such as liver, pancreatic, breast and bladder cancers, and is highly involved in regulation of tumour suppressors that downregulate AKT signalling by reducing the output of PI3K at the cell membrane 33, 38, 39. Our research suggests that CTT might directly regulate PTEN protein stability and thus negatively regulate AKT phosphorylation. Furthermore, PTEN siRNA increased the proliferation and invasion of 5637 cells. In addition, treatment of 5637 cells with PTEN siRNA significantly increased the expression of PI3K and p-AKT. Based on these results, we propose a novel mechanism by which CTT suppresses the proliferation and invasion of bladder cancer cells by activating PTEN and thus inhibiting the PI3K/Akt pathway. However, the manner in which PTEN is modified and its stability is regulated have yet to be elucidated.

**Figure 8:**
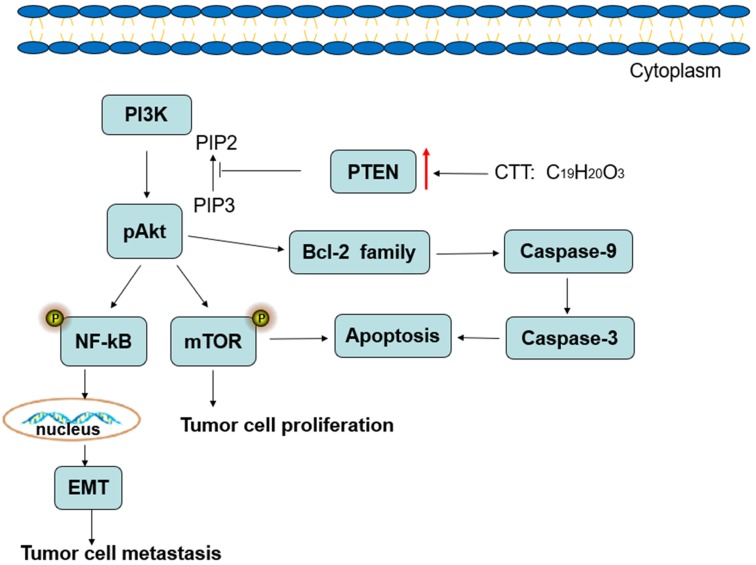
Diagram illustrating of the signaling pathways involved in CTT-mediated bladder cancer cell apoptosis.

In conclusion, the results of the present study demonstrate that CTT inhibits bladder cancer cell proliferation, migration and invasion and induces cell apoptosis, which may be associated with downregulation of PI3K/AKT/mTOR and NF-κB signalling pathway proteins, and this inhibition is mediated by the induction of PTEN (Figure 8). Therefore, our studies provide a therapeutic agent for inhibiting the growth and metastasis of bladder cancer.

## Supplementary Material

Supplementary figures.

## Authors' contributions

Yadong Liu, Fanlu Lin and Ruihua An: Study concept and design of the work. Yadong Liu, Fanlu Lin and Yaodong Chen: Acquisition of data. Yadong Liu and Jiannan Liu: Analysis and interpretation of data and drafting of the manuscript. Yadong Liu and Yinshan Jin: Critical revision of the manuscript for important intellectual content. Yadong Liu, Rui Wang: Statistical analysis. All authors read and approved the final version of the manuscript.

## Abbreviations

CTT: cryptotanshinone
NMIBC: non-muscle-invasive bladder cancer
PI3K: phosphatidylinositol-3 kinase
Akt: Protein Kinase B
mTOR: mammalian target of Rapamycin
FBS: fetal bovine serum
PBS: phosphate buffered saline
EMT: Epithelial-to-mesenchymal transition
