# Supplementary material for: Cryptotanshinone Inhibites Bladder Cancer Cell Proliferation and Promotes Apoptosis via the PTEN/PI3K/AKT Pathway
Source: J Cancer. 2020 Jan 1;11(2):488–99. doi: 10.7150/jca.31422 (PMC6930428; doi:10.7150/jca.31422)
Supplement: Supplementary file 1 — Supplementary figures. [file jcav11p0488s1.pdf]

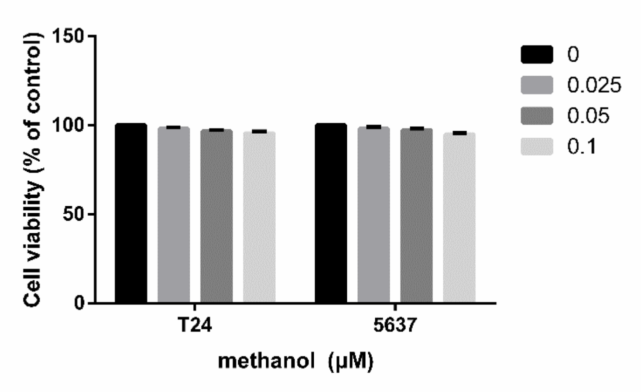

Figure S1. Bladder cancer cell lines (T24, 5637) were treated with various concentrations of methanol. Cell viability was measured by a CCK-8 assay.

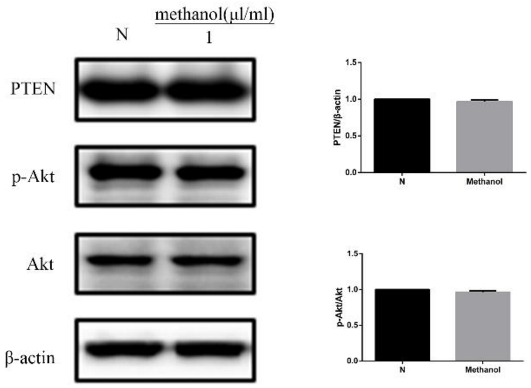

Figure S2. The expression of PTEN and p-Akt in 5637 cell treated with methanol diluted 1000 times.
